# Walking a mile in Grandma’s shoes - medical students’ evaluation of a very simple online aging game to enhance their understanding of older patients

**DOI:** 10.1186/s12877-022-03470-0

**Published:** 2022-11-16

**Authors:** Anne-Kathrin Geier, Stefan Lippmann, Antje Rau, Anne Schrimpf, Markus Bleckwenn, Tobias Deutsch

**Affiliations:** Department of General Practice, Medical Faculty, Leipzig University, Leipzig, Germany

**Keywords:** Aging game, Aging simulation, Online teaching, Undergraduate medical education, Geriatric medicine

## Abstract

**Background:**

Aging simulation games are established educational interventions to make older patients’ perspectives noticeable, raise awareness about their needs, and positively influence attitudes toward older adults. Due to the COVID-19 pandemic restrictions imposed on education, we replaced a classroom-based aging simulation course with a simple online equivalent. This consisted of short introductory screencasts, four downloadable Portable Document Format (PDF) files containing issue-specific audio and video links, quizzes, case studies, and prompts for reflection. We explored how well our self-directed simple online simulation succeeded in providing students with relevant insights and experiences, raising awareness about age-related difficulties, and enhancing understanding of older patients.

**Methods:**

In this cross-sectional study, an anonymous post hoc online survey was conducted among 277 5th-year medical students eligible for the course at the Leipzig University in May 2020. The questionnaire addressed overall course evaluations, assessments of the individual PDF components (working enjoyment, personal insights, professional learning gain, enhanced understanding, increased interest in working with older patients), and students’ main insights from the course (free text). Descriptive statistical and qualitative content analyses were performed.

**Results:**

The response rate was 92.4% (n = 256, mean age 25.7 ± 3.4 years, 59.8% women). Nearly all respondents reported that the course was well structured, easily understandable, and that processing was intuitive. The majority (82.8%) perceived the course as practice-oriented, 88.3% enjoyed processing, 60.3% reported having gained new professional knowledge, and 75.4% had new personal insights. While only 14.8% agreed that the online course could generally replace the real-world simulation, 71.1% stated that it enabled them to change their perspective and 91.7% reported enhanced understanding of older patients. PDF components containing audio and video links directly imitating conditions (visual or hearing impairment) were rated highest. Qualitative data revealed manifold insights on the part of the students, most frequently referring to aspects of professional doctor-patient interaction, knowledge about conditions and diseases, role reversal, and enhanced empathy.

**Conclusion:**

Simple online aging simulations may be suitable to provide students with relevant insights and enhance their understanding of older patients. Such simulations could be alternatively implemented in health professionals’ education where resources are limited.

**Supplementary Information:**

The online version contains supplementary material available at 10.1186/s12877-022-03470-0.

## Background

Aging simulations in the health sciences have been used for decades [1, 2] with the aim of sensitizing students to older patients’ functional limitations, positively influencing their attitudes toward caring for older patients, and enhancing empathy [3–6]. Aging simulations have been conducted with medical, nursing, and pharmacy students in different countries [7–10]. Although reviews have found mixed results of teaching interventions in geriatric medicine in general [4, 11] and educational role playing games in particular [12], single studies report significant increases in positive attitudes toward (caring for) older patients [3, 6, 10] and awareness of age-related difficulties [1, 13].

In Germany, as in many other countries worldwide, the geriatric population is constantly growing [14] and will constitute an important part of future physicians’ workload. This is especially true for general practitioners (GPs), who are the main healthcare providers for community-dwelling older people and will be confronted with a growing number of patients with multiple health problems [15, 16].

As we know, a ‘reluctance to treat too many old patients, chronic diseases and dying patients’ is among the reasons why medical students who seriously considered general practice (GP) careers in the first place finally decided against this option [17]. Studies investigating students’ attitudes toward geriatrics found that characteristics associated with this field (complex, chronically ill patients, long-term care, non-curable diseases) are not perceived as attractive [18]. However, older patients make up a significant proportion of patients in family practice [19]. To prepare medical students to care for older patients and to reduce potential barriers regarding a career in GP, the Department of General Practice of the Leipzig University established a course called Aging Simulation Game (‘Instant Aging’) in 2005. This 90-minute component is part of an interdisciplinary course in geriatric medicine. The course in geriatric medicine including the Aging Simulation Game is compulsory for all medical students in the 5th study year (of six). Students in small groups are “immersed” into four different physical impairments (hearing loss, visual impairments, tremor, joint constraints) with the help of selected devices. Prior to the COVID-19 pandemic, students used glasses to imitate cataract and other visual impairments, and listened to audio recordings simulating hearing impairment. Students were provided with an aging simulation suit with restrictions for the back and extremities. Transcutaneous electrical nerve stimulation devices were used to provoke a tremor in the students’ arms and hands while performing easy tasks like drinking water and brushing their hair. Each experience was followed by a discussion of their impressions and feelings as well as potential implications for their future medical activities.

Due to the COVID-19 pandemic that led to a complete lockdown with social distancing measures in Germany beginning in March 2020, the Leipzig University stopped classroom teaching in April 2020 (beginning of the summer semester). Teaching was completely switched to newly established online format. Accordingly, we designed an online-based self-directed version of the aging simulation game based on our long-lasting experience with classroom aging simulation. We intended to keep this online course as simple as possible to allow very easy imitation and adjustment of our ideas in other contexts. Nevertheless, the aim was to transfer the most meaningful and impressive classroom experiences to the students’ home office and to ensure comfort and ease of use.

It was hypothesized that simple online aging simulations could be easily established alternatives to resource-intensive classroom-based courses. This could be of special importance in low-budget or remote environments, targeting medical students as well as students and professionals from other social and health professions. Thus, we took the opportunity to explore comprehensively how our simple online aging simulation course (OASC) was adopted by the students and if the aims of raising awareness and enhancing understanding of older patients’ needs, as well as enhancing interest in geriatric care, could be achieved.

## Methods

### The online aging simulation course (OASC)

The OASC was designed by an interdisciplinary team of experienced academic researchers (physicians, psychologist, health services researcher). They contributed individual components based on their extensive previous experiences as instructors for the classroom-based aging simulation game. All components were distributed and refined after discussion with the whole research group. All researchers approved the final version.

For the online simulation course, four PDF documents were designed corresponding to the topics of the regular classroom aging simulation game. The resulting simple OASC included links to videos and audio files simulating different age-related disabilities as well as tasks for role reversal and reflection.

The self-explanatory PDF documents guided the students’ involvement with the following topics: hearing impairment/hearing loss, cataract/physical impairments in the context of medication intake, akinesia/rigor/joint constraints, and tremor. The documents contained links to preselected open-access videos (e.g., imitating progressive hearing loss and impaired visual acuity), tasks for individual engagement with the subject (“Find 5 medical aids that can compensate for the limited mobility of the limbs“) and reflection (“You are a person who is already very old. You have the cataract you just experienced [in the video…]. Note down at least 5 aspects that would most likely cause you difficulties.”). Students were also encouraged to use online resources for in-depth reading on the issues raised and to become familiar with medical aids. Standard solutions in the form of discussion points and thoughts expressed by students from previous years were included for some tasks. The scheduled duration was 80 minutes. An overview of the course content is provided in Table 1 and a translated version of the hearing part is in **Supplementary File 1** for illustrative purposes.

The material was available for download on an individually given day within a time period of one week in May 2020. The tasks could be worked on asynchronously by each student. An audio-visual slide cast was added that covered an introduction to the issue and instructions for the course and its evaluation. No offline or online synchronous teaching unit was scheduled due to limited time, technical concerns, and human resources. Students were provided with an email address in case questions occurred.

Participation in the course was mandatory for all regularly enrolled 5th -year students (n = 277). However, for technical reasons the actual participation in this evaluation study (downloading and processing of PDFdocuments) could not be controlled.

**Table 1:** Content of the online aging simulation course (OASC)

OASC part (PDF)	Tasks for the students	Audio/Video/Images	Solutions
Hearing impairment/hearing loss (*duration 20min)*	1. Think of situations […] in which you had to deal with hearing-impaired persons. What did you notice in your interactions? 2. Start the audio file and complete the (paper and pencil based) quiz, use ear plugs if available. 3. How did you feel during the quiz […]? 4. Watch the video simulating different stages of hearing impairment. 5. Imagine you are working as an intern on an internal medicine ward […]. What (simple) means can you use to ensure that important information […] is not “lost” due to impaired hearing function? List 8 points.	Audio file: Listening comprehension (quiz) with instructions on how to fill in a crossword puzzle, made difficult by strong background noise Video with sound: Different stages of hearing impairment Photo to support understanding of the task: Students from previous years with ear plugs performing the quiz	1. Answer key for the quiz 2. Typical discussion points and ideas raised by students from previous years’ courses were presented in tiny font size and could be enlarged
Cataract/physical impairments in the context of medication intake *(duration 20min)*	1. Watch the cataract video and put yourself in the shoes of an affected person. 2. Imagine you are a very old person with impaired vision and sensitivity/fine motor skills in your hands […daily 5 tablets plus drops … tablet sorter … time pressure…].Note 5 aspects that would probably cause difficulties in that situation. 3. Please note 5 aspects that could be considered by physicians regarding medication use in the home for geriatric patients.	Video: Cataract through the eyes of the patient Photo to support understanding of the task: Previous year-students wearing simulation glasses and gloves sorting and splitting tablets	1./2. Typical discussion points and ideas raised by students from previous years’ courses were presented in tiny font size and could be enlarged
Tremor *(duration 20min)*	1. Take the perspective of a patient with tremor in relation to the accomplishment of 3 daily tasks: Spooning soup, filling in crossword puzzles, cutting up tablets. 2. Watch the following 5 videos for illustration. 3. Please consider and research, if necessary, what things you should generally consider in the medical treatment of tremor patients with regard to their limitations	5 Videos: Patients suffering from tremor in everyday life situations/videos demonstrating typical medical aids.	N/A*
Akinesia/rigor/joint constraints *(duration 20min)*	1. With the impressions from the photo and the description in the text, try to understand the limitations of the affected patients when performing the following daily activities: Combing hair, climbing stairs, buttoning shirt 2. Watch the 3 following videos. 3. Find 5 medical aids that can compensate for the limited mobility of the limbs. 4. What modifications in patients’ homes could help facilitate the management of activities of daily living? Give 3 adjustments for the patient’s home.	3 Videos: Demonstration of Aging suits/aging games in different contexts Photo: Classroom impressions, student with aging suit	N/A*

*N/A = not applicable

### Sampling and design

Data were collected in May and June 2020 at the Leipzig University. For the online survey, the evaluation software Evasys was used (Evasys, Electric Paper Evaluationssysteme GmbH, Lüneburg, Germany). To ensure participation only once per person, a personalized link was sent by email to all 5th -year medical students who were eligible for the course one day before the first students were enrolled. The emails were sent by the central administration of the medical faculty resulting in completely anonymous data sets without identifiers for the researchers. The evaluation link remained active for four weeks and a reminder was sent one day after the last students completed the course. Students were informed about the background of the study and the anonymity of the survey.

### Questionnaire

A self-designed questionnaire was developed by a multidisciplinary team of experienced scientists including physicians, a psychologist, and a health services researcher. It contained questions addressing sociodemographic information, professional orientation, and computer literacy to describe the sample. Further questions related to the overall evaluation of the online course (overall assessments with regard to content and technical aspects as well as perceived benefit). Finally, it contained items to assess the individual PDF components regarding variables of central interest: working enjoyment, personal insights, professional learning gain, enhanced understanding of older people, and enhanced interest in working with older patients. Answer format was a forced-choice, 4-point Likert-type scale, which did not allow neutral responses. In additional free text questions, students were asked to briefly state their key insights from the OASC. An English translation of the questionnaire used in this study is provided in **Supplementary File 2**. To ensure suitability and face validity the questionnaire was pre-tested online with two available students who were not eligible for the course representing the target group. In addition to general feedback from participants, pre-testing was guided by the Concurrent Think Aloud method, which resulted in minor adjustments in the final version.

### Data analysis

Data were analyzed using IBM SPSS 25 (Armonk, NY, USA) for Windows. The analysis was mainly descriptive based on the single items (questionnaire did not contain scales based on multiple items). Continuous variables are presented as mean ± standard deviations (SD) and frequencies are presented as %_valid_ (n_absolute_/n_valid_) considering missing values for individual items. Frequencies were compared using chi-square test.

The qualitative raw data of the free text answers were analyzed following a deductive-inductive approach. In a first step, relevant aims and issues were identified from literature [4, 11, 12, 20, 21] by the first author (GP trainee) as potential master categories. In a second step, the first author and an additional health scientist (based in health services research, both experienced with the aging simulation course) refined and complemented categories independently from each other following an inductive approach according to Mayring’s qualitative content analysis [22] and including all available material. The resulting category systems were compared, and consensus was found for all differences. Subsequently, the material was independently assigned to the final category system by both raters. Assignments were compared, and consensus was again found for all discrepancies. Applicable categories were used only once per person.

To assess the reliability of the results, a third previously uninvolved rater (physician) allocated the raw data once again to the final category system. Agreement with the first two raters’ consensus was 73.1%, which can be considered as acceptable due to the high number of categories and the nature of the material (brief free text notes). Discrepancies were discussed among all three raters and a final allocation determined. In a last step, absolute and relative category frequencies were calculated.

## Results

### Sample characteristics

In total, 277 students were requested to take part in the course, of which 256 participated in the evaluation (response rate = 92.4%). The non-response of 7.6% may be due to non-participation in the course as well as in the evaluation. The respondents’ mean age was 25.7 ± 3.4 years (Min: 22, Max: 43, Median: 24 years) and 59.8% (152/254) were female. Detailed sample characteristics including socio-demographics as well as students’ current career preferences are shown in Table 2.

**Table 2:** Sample characteristics and current career considerations

Variable	valid (N)*	N (%)**
Age [mean ± SD]	254	25.7 ± 3.4
Female	254	152 (59.8)
Mainly grown up in	255	
big city		87 (34.1)
small town		87 (34.1)
rural area		81 (31.8)
Pre-existing concluded education in a medical vocational education	255	54 (21.2)
To become a general practitioner is	255	
the favored career option		27 (10.6)
an imaginable career option		162 (63.5)
no career option		66 (25.9)
Future work in ambulatory care is	256	
the favored career option		104 (40.6)
an imaginable career option		145 (56.6)
no career option		7 (2.7)
Working self-employed in the future (own practice) is	256	
the favored career option		81 (31.6)
an imaginable career option		159 (62.1)
no career option		16 (6.3)

* Ns vary due to missing values

** unless otherwise indicated

### Computer literacy and device usage

In the self-assessment of their computer literacy, 34.0% (87/256) rated themselves ‘very fit’ in dealing with computers, 55.1% (141/256) ‘rather fit’, 10.5% (27/256) ‘rather not fit’, and 0.4% (1/256) ‘absolutely not fit’. Women stated slightly more frequently that they were rather or absolutely not fit (women 14.5% [22/152] vs. men 5.9% [6/102], p = 0.032).

Most students (76.6% [196/256]) used laptop computers for the course, while 16.0% (41/256) used tablets, 12.1% (31/256) used desktop computers, and 5.5% (14/256) used smartphones (multiple answers were possible).

### Quantitative evaluation of the online course and its components

**Fig. 1:**
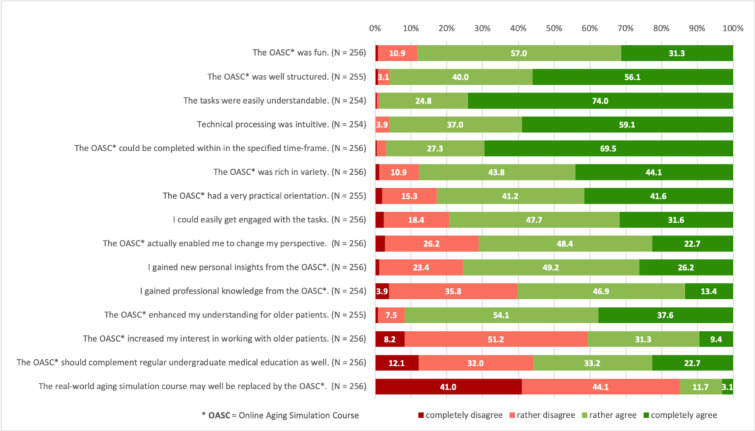
Students’ general post hoc assessments of the OASC*

Results of the assessments of the OASC in general are presented in Fig.1.

The majority of the students evaluated the course positively regarding structure, understanding, processing, fun while completing, perceived variety, and practice orientation. Although more than 85% of the respondents disagreed that the online course may well replace a conventional real-world instant aging game, more than 90% reported that the online course enhanced their understanding about older patients. Most students reported having gained personal (75.4%) and professional (60.3%) insights and that the course was successful in enabling a perspective change (71.1%). Four out of ten participants stated that the course increased their interest in working with older patients.

**Fig. 2:**
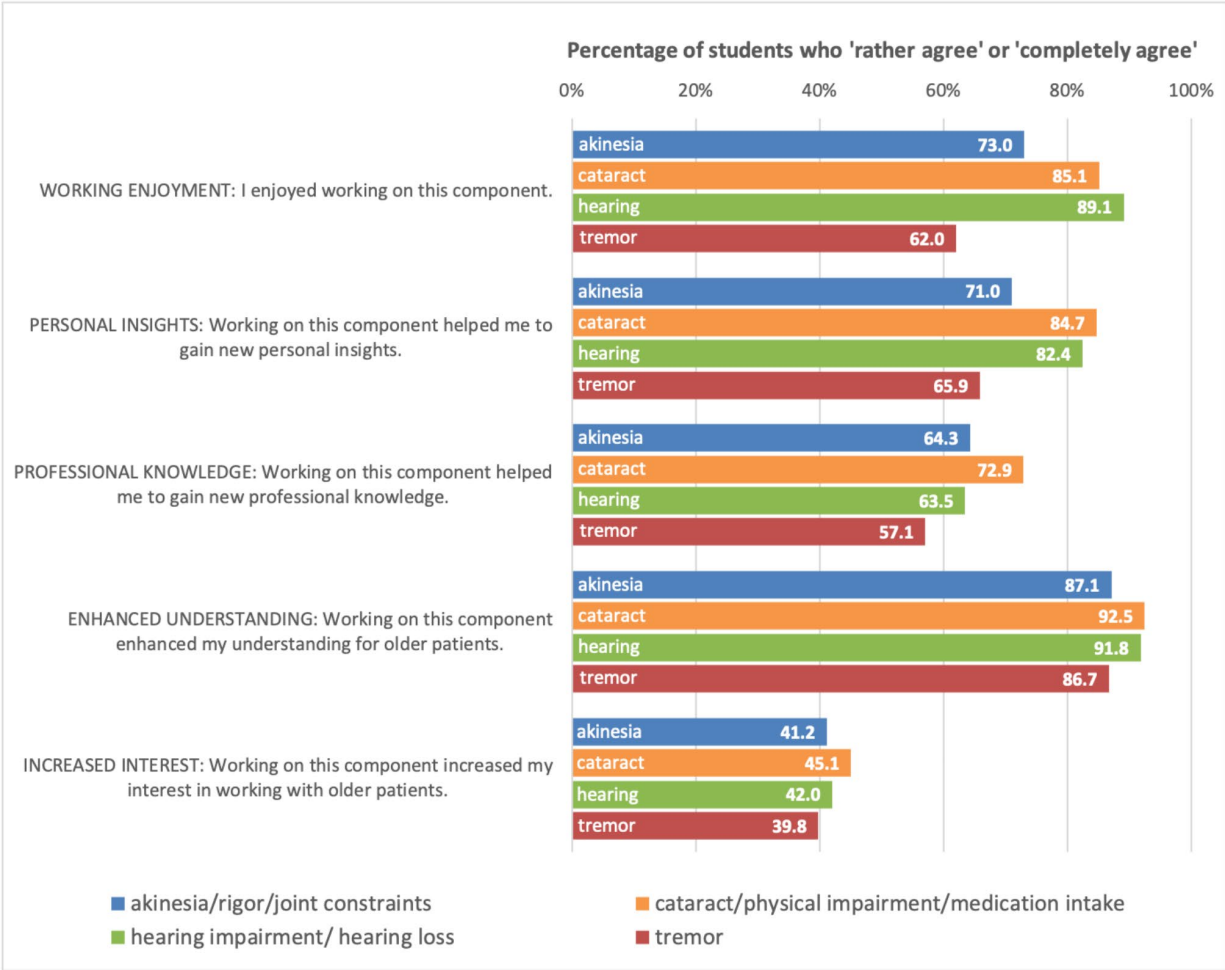
Students’ assessments of the four individual PDF components of the online aging simulation course (OASC)

Results of the individual component assessments are shown in Fig.2.

Students were asked to assess the four individual PDF components of the online course separately regarding the following five aspects: fun while completing the task, gain of personal insights, gain of professional knowledge, enhanced understanding of older patients, and increased interest in working with older patients.

All individual components were perceived as particularly helpful for enhancing the understanding of older patients by an overwhelming majority (> 85%) of the evaluation participants. The PDF components dealing with ‘hearing impairment/hearing loss’ and ‘cataract/physical impairments in the context of medication intake’ received higher agreements than akinesia/rigor/joint constraints and tremor in the categories working enjoyment, personal insights, enhanced understanding and increased interest in working with older patients.

### Qualitative analysis of the evaluation participants’ key insights from the online course

Altogether, 235 students provided brief free text responses regarding their main insights from the OASC. The respective responses contained altogether 516 delimitable statements, between 1 and 5 (mean 2.2 ± 1.1, median 2) per student. The insights expressed by the evaluation participants could be subsumed to the following major categories: Information on professional interaction with geriatric patients, including background knowledge on specific issues, specific knowledge about geriatric conditions and diseases (symptoms, therapies, coping strategies), statements on the methods used: experience of role reversal, others (e.g. statements on own aging process and physical power), statements about (enhanced) empathy and understanding toward older people, and statements on changed attitudes toward older people. Table 3 provides a detailed overview of all major categories and subcategories including absolute and relative frequencies of students who expressed respective statements, and example quotes.

**Table 3:** Results of the qualitative analysis of students’ free text answers

“Please briefly specify your main insights from the online aging simulation course: ____”*				
**Major Categories**	**Subcategories**	**Example quotes**	**Number of students** **N**	**Percentage of students** **%**
Information on professional interaction with geriatric patients, including background knowledge on specific issues *Total of single statements assigned to this major category*: 341	Patients’ everyday life, living environment, and family	“Older people have important impairments in everyday life.” (30 years, male) “Taking older peoples’ problems more serious, because even simple situations in everyday life can cause difficulties.” (24 years, female)	71	30.3
	General statements on how to deal with older people (professionally)	“In general, and more specifically as a doctor, you should have sympathy!” (23 years, female) “Patience and understanding are important in the interaction with older people.” (33 years, male) “As a ‘young and healthy person’, one should have patience and sympathy with older people […].” (25 years, female)	67	28.6
	General difficulties associated with the process of aging	“More impairments in old age than expected, it is not only pain or loss of power but many things together.” (30 years, female) “Until now, I underestimated disabilities occurring with old age […].” (25 years, female)	55	23.5
	(Provision of) medical aids	“Many medical aids on the market, as a doctor you have to deal with the assortment to be able to help.” (23 years, female)	55	23.5
	Doctor-patient communication	“Taking time is a very important aspect for the communication with older patients.” (24 years, female)	44	18.8
	Drug prescription and intake	“What can cause difficulties in taking drugs and how one can react to that as a doctor.” (24 years, female)	34	14.5
	Geriatrics as a medical specialty - importance and appreciation	“Geriatrics is a very important point in medicine and therefore in our education.” (24 years, male)	8	3.4
	(Importance of) prevention	“Preserving the senses and physical resilience of the old is firstly an enormous gain in quality of life and secondly a relief for the health care system.” (24 years, male)	4	1.7
	Psychological impairments in older people	“Physical and mental decay reinforce each other.” (30 years, male)	3	1.3
Specific knowledge about geriatric conditions and diseases (symptoms, therapies, coping strategies) *Total of single statements assigned to this major category*: 89	Hearing impairment/hearing loss	“I never realized how difficult hearing is for patients when ambient noise is high.” (30 years, female) “Hearing loss is stressful.” (24 years, male)	37	15.8
	Visual impairment	“Visual impairment in cataract significantly more limiting than thought.” (25 years, female)	19	8.1
	Movement disorders: tremor	“A tremor can strongly affect the activities of daily life, in this practical course very helpful and innovative aids were introduced (e.g., special tremor eating spoon).” (24 years, female)	14	6.0
	General sensory impairments	“Decreased sensory perceptions make contact with the outside world difficult and cause confusion.” (24 years, female)	9	3.8
	Movement disorders: others, combined, or non-specific	“Akinesia, rigor, and joint restrictions can badly limit the patient’s mobility, a doctor should prescribe adequate medical aids (e.g., wheeled walkers).” (24 years, female)	8	3.4
	Movement disorders: walking disabilities	“Difficulty of moving around […]” (25 years, male)	2	0.9
Statements on the methods used: experience of role reversal	*(No subcategories)*	“The videos made it a lot easier for me to imagine an older person’s life with cataract.” (23 years, female) “Change of perspective can be useful.” (23 years, male) “Role reversal is worthwhile to raise awareness for the situation of older patients and associated problems […].” (23 years, female)	41	17.5
Others *Total of single statements assigned to this major category*: 23	Statements on own aging process and physical power	“One does not appreciate healthiness as long as one is healthy.” (24 years, female) “[…] We will all be old one day and life should be as easy as possible for everyone.” (23 years, female) “One does not think about problems in old age, takes everything for granted.” (23 years, female).	20	8.5
	Pleasant aspects of aging	„You do not need to be afraid of old age, there are many ways to cope well in everyday life […]” (23 year, female)	1	0.4
	Not assignable to any category	“General practice is my thing” (23 years, female)	2	0.9
Statements about (enhanced) empathy and understanding toward older people	*(No subcategories)*	“Main insights are among all a more precise understanding and more empathy for problems in older people’s everyday life.” (24 years, male) “Enhanced empathy for older people with impairments related to old age.” (age not indicated, female)	20	8.5
Statements on changed attitudes toward older people	*(No subcategories)*	“It is worthwhile investing time in the treatment of older people.” (28 years, female) “Respect for old age and people who actively maintain a social life despite their impairments.” (28 years, female)	2	0.9

*Major categories and subcategories, sorted in descending order by frequency of mentioning; n_valid_=235 students)

### Information on professional interaction with geriatric patients

Most statements in this major category referred to new insights regarding older patients’ everyday lives. There were statements recognizing the difficulties occurring with the process of aging, and about professional or general implications for dealing with geriatric patients. Some statements more specifically referred to professional aspects such as communication, prevention, means of support, and medication.

In this category, we additionally subsumed the many statements that referred to “understanding” in an impersonal way and when a reference to individual feelings and processes was not clearly stated, such as “In general, and more specifically as a doctor, you should have sympathy!” (23 years, female).

### Specific knowledge about geriatric conditions and diseases (symptoms, therapies, coping strategies)

Many statements referred to the specific conditions presented in the aging simulation course such as hearing loss/hearing impairment and visual impairment, and their symptoms from the patient’s perspective. They also targeted strategies to approach and treat patients with the respective conditions.

### Statements on the methods used: experience of role reversal

Some students referred to their experiences with, the effects of, and their assessment of the methods used. While most statements were positive, some also criticized the methods and referred to the limits of the digital experience.

### Others

This category was dominated by statements indicating a reflection with the students’ own aging process and (physical) abilities as well as comparisons between their own perceptions and that of older people.

### Statements about (enhanced) empathy and understanding toward older people

We assigned few statements to this category that explicitly indicated a gain in empathy and understanding in the students.

Only two statements indicated *changed attitudes* toward (working with) older people.

## Discussion

### Summary of the main results

Because classroom-based aging simulation was not possible in the beginning of the COVID-19 pandemic, a simple OASC based on interactive PDF documents for 5th-year medical students was established. Despite the simple technical implementation, most of the students reported that they enjoyed working on the course. They perceived it to be rich in variety and practice-oriented. The majority reported having gained new personal insights and professional knowledge due to the course and nearly all evaluation participants in our study confirmed an enhanced understanding of older patients. Main insights from the course were many and most frequently referred to aspects of professional interaction with geriatric patients, knowledge about geriatric conditions and diseases, role reversal, and enhanced empathy.

### Interpretation of the findings and literature comparison

#### Computer literacy, device usage, and overall evaluation

The vast majority of students in our evaluation reported being fit or rather fit in dealing with computers. This corresponds to our finding that nearly all participants confirmed intuitive technical processing. As insufficient information technology (IT) skills have been identified as important barriers to successful digital learning [23], these findings are confirmative and we are confident that the course was constructed according to the students’ technical means and skills. Most respondents used laptops for the course. The use of PDF documents might have limited the use of smaller devices and mobile phones. On the one hand, using smartphone compatible e-learning applications in undergraduate education might be important, particularly in less resource-rich environments. On the other hand, easy to establish material on the part of the institution is critical to counter the challenge that “e-learning is a time-, cost- and labour-intensive approach” [23], which was not the case for our course.

Overall, the student-participants reported that they enjoyed working on the course. There was a very high satisfaction regarding formal aspects such as understandability of the tasks, intuitive processing, course structure, and timing. Furthermore, the students perceived the course content to be rich in variety and practice-oriented. It can thus be concluded that the course was user friendly, convenient to work on, well-structured, and convincing in terms of content.

#### Raised awareness, understanding, and empathy

Over 90% of the participating students rather or completely agreed that the course enhanced their understanding of older patients. This was also the statement that scored highest in the evaluation of the single components. Our qualitative analysis drew a more precise picture: while few students directly reported a gain of understanding as their main insight, many statements that were primarily assigned to other categories reported insights into the patients’ experiences and feelings. Students expressed their conclusions drawn from respective impressions and their implications for future work with geriatric patients. The authors find that even simple educational means could attain goals of raising awareness and enhancing understanding of older people in most of the participating students.

When analyzing the data, the authors found it difficult to distinguish between the terms “*understanding*”, which was finally applied to a primarily cognitive process (get insights, be aware, take in mind) and “*empathy*”, which has received important attention as a potential outcome in medical (and more specifically geriatric) education. *Empathy* has been referred to as “the consequences of perceiving the feeling state of another as well as the capacity to do so accurately”[24] and both its cognitive and emotional components have been emphasized in the literature. However, in the context of healthcare, empathy has been more clearly defined by Hojat and colleagues as an “attribute that involves an *understanding* (rather than feeling) of pain and suffering of the patient, combined with a capacity to *communicate* this understanding, and an *intention to help”* [25]. Batt-Rawden and colleagues summarized interventions to increase and maintain empathy in undergraduate medical students in their review and concluded that interventions can “successfully cultivate empathy”[21] but equally criticized methodological flaws and the elusive nature of an operational definition of empathy [21]. Similar results have been reported in a more recent review by Bearman and colleagues, who stated that interventions that “ask the learner to act in the role of patient may be more effective in developing empathy” [20]. In this study, some participating students explicitly mentioned a gain of empathy in their free text answers, though the authors believe that what they had in mind might not correspond absolutely to the theoretical concepts discussed above. In (subjective) synopsis, the results of the present study indicate a possible positive change with regard to empathy. However, this would need to be verified in studies using more appropriate methods. While this study did not explicitly measure “*attitude”*, it was astonishing that only two participants made statements that could be interpreted as changed attitude in the qualitative analysis. Since validated tools exist to measure attitudes toward older adults [26–28], this aspect has received much attention in literature. Two recent reviews included a wide range of geriatric interventions of whom at least some were effective in changing attitudes, though overall results were mixed [4, 11]. Interventions that included an empathy-building component in contrast to knowledge-only interventions seemed to be more effective in positive attitude changes[4] and Tullo and colleagues concluded that “the simulation approach that seems most beneficial is one that asks the learners to literally stand in patients’ shoes” [11]. This seems even more plausible as similar observations have been reported for “empathy” as discussed above. More precisely, positive attitude change has been reported for three studies evaluating courses using means similar to what we usually do (“Aging simulation”/“Aging game” as opposed to seminars and clinical visits) and which formed the basis for our digital intervention [3, 5, 6]. Therefore, the authors raise for discussion that this study may have had a greater effect on changing attitudes than it was able to show in the analyses. An important implication for further evaluation is thus to integrate validated and well-established tools as outcome measures.

#### New knowledge gained

Previous findings underlined that online learning is at least as good as offline learning in terms of knowledge transfer [29, 30] but has important advances in terms of flexibility and accessibility. This intervention was not primarily designed to transfer *knowledge* in a systematic and comprehensive way. However, more than 60% of the students stated that they had gained professional knowledge. This refers to the whole course as well as the four individual components, except for tremor, which scored slightly lower. The qualitative analysis revealed in more detail that what was labeled as “knowledge” often referred to very specific aspects of conditions and diseases (“how hearing loss sounds like for the affected patient” in contrast to pathophysiological and anatomical basics of diseases). Furthermore, students got in contact with domains that obviously had not played an important role in their prior studies and this raised their interest. Some of the linked videos contained information on medical aids (e.g., spoon instead of fork used for people suffering from tremor) that were presented in a rather funny way and the research team noticed that this must have impressed the students much more than other (maybe more important) details. We suggest not underestimating the chance of knowledge transfer in a teaching format primarily designed for self-awareness and drafting materials and choosing external sources accordingly.

#### Increased interest in dealing with older patients

A further important result is that around 40% of the students agreed that the OASC enhanced their interest in working with older patients. Besides the general importance of positively influencing the interest of young doctors in working with a growing subgroup of patients, this finding is of relevance regarding the recruitment of GPs. Previous research showed that reservations against working with older patients contributed to the rejection of GP as a career choice in graduates who had seriously considered becoming GPs [17]. Consequently, undergraduate interventions successfully enhancing the interest in working with older patients may help positively influence the image of GP and the consideration of a GP career. This is of particular importance due to the growing shortage of GPs in Germany as well as in many countries worldwide [31–35], particularly affecting rural regions [36–38]. Thus, there is an urgent need to attract more future physicians to the specialty to maintain an area-wide primary care supply close to home for all patients. However, it was noted that only about 40% of the students reported an enhanced interest, while the majority did not. Regardless of the reasons (student characteristics, baseline interest in working with older people, course content, methods, etc.) this leaves room for improvement and further investigation on the determinants of failure and success.

#### Insights from individual PDF component evaluations

Based on the evaluations of the four individual PDF components used in this course, the authors find that a change of perspective worked best for the components “hearing impairment/ hearing loss” and “cataract/physical impairments in the context of medication intake” where audio files and videos imitated central conditions. Consequently, students could immerse themselves in the patients’ experience. Both components scored highest in almost all categories including enjoyment, personal insights, gain of professional knowledge, and understanding of older people. This was confirmed by the qualitative analysis of the students’ main insights, where hearing loss and visual impairment were the most frequently mentioned conditions that were impressive or helpful. Although students were faced with videos demonstrating the other conditions (tremor, joint constraints), they remained in observer status instead of “walking in the patient’s shoes” themselves. Future OASCs should focus on conditions that are easy to experience directly (e.g., by digital means) and/or enable the immersion into other conditions. As a result of these findings, students completing the OASC at Leipzig university in the following year 2021 were provided with detailed instructions for “do-it-yourself” ideas to imitate movement restrictions with the help of bandages and sports pads (before returning to the classroom in 2022).

### Comparison with online and classroom interventions

Several studies have investigated digital teaching interventions in medicine with great success. However, some of them seem to be technically and in terms of preparation and manpower quite demanding and thus not necessarily suitable for a great number of students or in resource-poor environments. For example, Halton and Cartwright evaluated an intervention called “In Their Shoes®” that is delivered via smartphone application. It runs over two days and provides students with an avatar, including several live role plays [39]. Other digital formats include three-dimensional (3-D) learning environments in virtual worlds such as Second Life [40, 41], and 3-D glasses [42]. It seems especially true for virtual reality applications that they have pros and cons that deserve intensive evaluation and discussion, not only in terms of outcome but also regarding accessibility, manpower, and financial resources in general and in specific contexts [43, 44].

Nevertheless, the vast majority of the students in this study stated that the simple OASC could not generally replace the classroom-based real-world experience (that they had not had the possibility to experience themselves but might have heard about from older students). The authors agree that this is true especially for components that require technical devices and sophisticated materials such as the aging simulation suit. Furthermore, sharing and discussing the experience with peers is surely increasing learning enjoyment and learning gain. However, with small adjustments we believe that digital components such as videos could be a suitable complement and enrich conventional courses.

### Strengths and limitations

This study addresses an innovative topic and has relevant implications for undergraduate medical education and the education of health professionals in general. The very high response rate of more than 90% indicates representativeness of the sample. The mixed methods approach and the integrated interpretation of quantitative and qualitative data strengthen the explanatory power of the results.

Learning gain and understanding of older people were self-rated and are therefore prone to bias. Students could have overestimated their professional knowledge and skewed their answers to fit social desirability. This is especially true for the beginning of the lockdown period, because of the difficult circumstances the students went through. The evaluation may have been particularly benevolent against the background of a “better than nothing” attitude that the students experienced at this time.

Due to the unprecedented circumstances, no validated instruments were used to measure outcomes and no pre-post-analysis was performed, which limits the validity of the findings. Additionally, the self-designed questionnaire hinders the comparison of the results with existing interventions online or offline. However, triangulating the questionnaire with the responders’ free text answers shows a high level of consistency and confirms the students’ engagement with many issues in geriatric medicine. That is quite affirmative, because no other mechanisms to monitor the students‘ investment in the course were used, apart from adequate numbers of clicks on the download page.

The evaluation of the qualitative analysis was fraught with ambiguity due to the response format (key points). The used methodology (three raters, acceptable agreement) attempted to minimize uncertainties in order to arrive at a reliable result, although we assume that the overall statement does not change significantly due to imprecision.

As a further limitation, it should be noted that the results represent short term effects. This is due to the study design and a well-known point for criticism [45]. Whether results are stable and if respective interventions have positive impacts on behavior and clinical outcomes in the future could be a subject of further investigation.

## Conclusion

This study shows that a very simple OASC that can be established with very low effort and resources can have remarkable effects. Despite methodological limitations, the authors conclude that it can enhance medical students’ awareness and understanding of older patients and their needs and provides relevant personal and professional insights and knowledge. Furthermore, it can increase the interest of a certain proportion of students in working with older patients. Medical students welcome the learning content and can experience role reversal and change of perspective with very basic means. Considering the cost-benefit ratio regarding the simplicity of our online intervention and its reported effects on the students we see valuable implications for undergraduate medical education as well as the education of professionals in health and social sciences in general. Simple online aging simulations can be easily implemented with low efforts and costs and could be a recommendable ‘better than nothing’ alternative when the establishment of resource-intensive real-world simulations or other more cost-intensive online interventions is not feasible. This study could be a starting point for the further development of this sort of ‘simple aging simulations’ and further research on the topic, addressing possible long-term effects and using more sophisticated study designs.

## Electronic supplementary material

Below is the link to the electronic supplementary material.

Supplementary Material 1. English translation of the online aging simulation course, course part “Hearing impairment/hearing loss"

Supplementary Material 2. English translation of the questionnaire used for the study

## Data Availability

The datasets used and/or analyzed during the current study are available from the corresponding author on reasonable request.

## List of abbreviations

3-D: Three-dimensional.
GP: General Practice.
GPs: General Practitioners.
IT: Information Technology.
N/A: Not applicable.
OASC: Online Aging Simulation Course.
PDF: Portable Document Format.
