# Supplementary material for: Walking a mile in Grandma’s shoes - medical students’ evaluation of a very simple online aging game to enhance their understanding of older patients
Source: BMC Geriatr. 2022 Nov 16;22:865. doi: 10.1186/s12877-022-03470-0 (PMC9667442; doi:10.1186/s12877-022-03470-0)
Supplement: Supplementary file 1 — Supplementary Material 1. English translation of the online aging simulation course, course part “Hearing impairment/hearing loss" [file 12877_2022_3470_MOESM1_ESM.docx]

**Supplementary file 1:**

**English translation of the online aging simulation course,**

**course part** “**Hearing impairment/hearing loss"**

*Explanations added for the reader are in italics.*

**Listening station**

Estimated time to complete this station: 20 minutes.

This station deals with the simulation of age-related hearing loss (presbyacusis).

*At this point, a photo had been inserted showing students with earplugs performing the task described below.*

You are asked to take the perspective of a patient with hearing loss. The main focus is on the limitations in everyday life and the resulting consequences for the medical care of these patients.

To begin with, think of situations, e.g. in a clinical clerkship, or even in your family, in which you had to deal with hearing-impaired persons. What did you notice in your interactions with this person?

-

-

-

In the following listening example with quiz we have tried to make the restrictions more comprehensible for you, although a 100% realistic representation is of course not possible.

1. Print out the page with Fig.1.

2. Have a pencil ready.

3. To further limit hearing, you can (if available) additionally put on or use (noise protection) headphones or earplugs.

4. Start the audio sample and follow the instructions contained therein. Please open the separately attached file "*Audio file*"

Use Fig.1 for the quiz. (First you have to guess which of the following words is meant, then look for the letter described. Pay attention to indications like "...letter from behind" or "...letter from the front". Put the letters one by one in the tally sheet).

5. What is the solution sentence?

*(Audio file contains instructions from a speaker to fill in the quiz, e.g. “find the word that describes a famous men, note down the third letter from behind”. The speaker’s voice is accompanied by important background noise such as in a café or at the railway station. Students usually find it difficult to follow in the beginning but adapt to the situation in the course of the tape)*.

| Fig. 1: Please print | |
| --- | --- |
| Elephant | Altenburg |
| Lettuce | Brazil |
| Lawyer | Desk lamp |
| Rhinorhoea | John Fitzgerald Kennedy |
| Suspenders | spoiled |

How did you feel during the quiz? What did the quiz do to you?

-

-

-

-

Here you can find another short video Link on the topic. *(Link to online video illustrating hearing loss).*

You should now have an idea of what environmental variables can further limit a hearing impaired patient.

Imagine you are working as an intern on an internal medicine ward and have to discuss the procedures after discharge from hospital with your 80-year-old patient Mrs. Meyer with presbyacusis.

What (simple) means can you use to ensure that important information about, for example, taking tablets is not "lost" due to her impaired hearing function? List 8 points:

-

-

-

-

-

-

If you would like to know what your fellow students usually tell us after this self-experience, simply enlarge the following mini-text (zoom in or copy to Word and resize text):

Solution for the quiz: I NEED A HEARING AID

Some examples of solutions or ideas that we collected with students in previous years for the last task with patient Mrs. Meyer:

Address hearing limitations openly and sensitively to take away patient's fear of dealing with her hearing limitations, allow sufficient time for the conversation, if possible take patient into a separate room or send other patients out of the room for a short time, if possible no disturbances by nursing/physiotherapy.... Close the windows, turn off the radio/TV, sit opposite the patient, use a hearing aid if available, speak slowly and clearly, write down or draw important information (medication schedule...) and give it to the patient, pause briefly after important facts and ask whether the patient has understood everything. Bring in a trusted person of the patient, e.g. daughter/son/spouse, and discuss topics together, also note all important information in the discharge letter.
