# Supplementary material for: Walking a mile in Grandma’s shoes - medical students’ evaluation of a very simple online aging game to enhance their understanding of older patients
Source: BMC Geriatr. 2022 Nov 16;22:865. doi: 10.1186/s12877-022-03470-0 (PMC9667442; doi:10.1186/s12877-022-03470-0)
Supplement: Supplementary file 2 — Supplementary Material 2. English translation of the questionnaire used for the study [file 12877_2022_3470_MOESM2_ESM.docx]

**Supplementary file 2:**

**English translation of the questionnaire used for the study**

| **Socio-demographic information** | | | | | | | | | | | | | | | | | | | | | | | | | | | |
| --- | --- | --- | --- | --- | --- | --- | --- | --- | --- | --- | --- | --- | --- | --- | --- | --- | --- | --- | --- | --- | --- | --- | --- | --- | --- | --- | --- |
| Age: |  | \|__\|__\| years | | |  | Sex: | | | | ○ | | | | male | | | ○ | | female | | | | | | | | |
| Semesters (total) | | | \|__\|__\| semesters | |  |  | | | |  | | | |  | | |  | |  | | | | | | | | |
| Where did you mainly grow up? | | | | | | | ○ | big city | | | | ○ | | | | small town | | | | ○ | | rural area | | | | | |
| I have a (concluded) qualification in a medical vocational education. | | | | | | | | | ○ | | Yes, ________________________ | | | | | | | | | | | | | | ○ | No | |
| How do you assess your computer skills? | | | | | | | | ○ very fit | | | | | ○ rather fit | | | | | ○ rather not fit | | | | | | ○ absolutely not fit | | |  |
| **Career preferences** | | | | | | | | | | | | | | | | | | | | | | | | | | | |
| To become a general practitioner is … | | | | ○ | the favored career option | | | | | | | ○ | | | an imaginable career option | | | | | | ○ | | no career option | | | | |
| Future work in ambulatory care is … | | | | ○ | the favored career option | | | | | | | ○ | | | an imaginable career option | | | | | | ○ | | no career option | | | | |
| Working self-employed in the future (own practice) is … | | | | ○ | the favored career option | | | | | | | ○ | | | an imaginable career option | | | | | | ○ | | no career option | | | | |

| **General assessment of the online aging simulation course** | | | | | | | | |
| --- | --- | --- | --- | --- | --- | --- | --- | --- |
| Which electronic device did you use for the online aging simulation course? *(Multiple answers possible)* | ○ laptop computer | | ○ desktop computer | | ○ tablet computer | | ○ smartphone | |
| **To what extent do you agree with the following statements?** | | *I completely agree* | | *I rather agree* | | *I rather disagree* | | *I completely disagree* |
| The online aging simulation course was fun. | | ○ | | ○ | | ○ | | ○ |
| The online aging simulation course was well structured. | | ○ | | ○ | | ○ | | ○ |
| The tasks were easily understandable. | | ○ | | ○ | | ○ | | ○ |
| Technical processing was intuitive. | | ○ | | ○ | | ○ | | ○ |
| The online aging simulation course could be completed within in the specified time-frame. | | ○ | | ○ | | ○ | | ○ |
| The online aging simulation course was rich in variety. | | ○ | | ○ | | ○ | | ○ |
| The online aging simulation course had a very practical orientation. | | ○ | | ○ | | ○ | | ○ |
| I could easily get engaged with the tasks. | | ○ | | ○ | | ○ | | ○ |
| The online aging simulation course actually enabled me to change my perspective. | | ○ | | ○ | | ○ | | ○ |
| I gained new personal insights from the online aging simulation course. | | ○ | | ○ | | ○ | | ○ |
| I gained professional knowledge from the online aging simulation course. | | ○ | | ○ | | ○ | | ○ |
| The online aging simulation course enhanced my understanding for older patients. | | ○ | | ○ | | ○ | | ○ |
| The online aging simulation course increased my interest in working with older patients. | | ○ | | ○ | | ○ | | ○ |
| The online aging simulation course should complement regular undergraduate medical education as well. | | ○ | | ○ | | ○ | | ○ |
| The real-world aging simulation course may well be replaced by the online aging simulation course. | | ○ | | ○ | | ○ | | ○ |

| **Assessment of the online aging simulation course by components** | | | | |
| --- | --- | --- | --- | --- |
| **A – Working enjoyment** | | | | |
| **I enjoyed** working on this component**:** | *I completely agree* | *I rather agree* | *I rather disagree* | *I completely disagree* |
| … Akinesia/rigor/joint constraints | ○ | ○ | ○ | ○ |
| … Cataract/physical impairments in the context of medication intake | ○ | ○ | ○ | ○ |
| … Hearing impairment/hearing loss | ○ | ○ | ○ | ○ |
| … Tremor | ○ | ○ | ○ | ○ |
| **B – Personal insights** | | | | |
| Working on this component helped me to gain **new personal insights:** | *I completely agree* | *I rather agree* | *I rather disagree* | *I completely disagree* |
| … Akinesia/rigor/joint constraints | ○ | ○ | ○ | ○ |
| … Cataract/physical impairments in the context of medication intake | ○ | ○ | ○ | ○ |
| … Hearing impairment/hearing loss | ○ | ○ | ○ | ○ |
| … Tremor | ○ | ○ | ○ | ○ |
| **C – Professional knowledge** | | | | |
| Working on this component helped me to gain **new professional knowledge:** | *I completely agree* | *I rather agree* | *I rather disagree* | *I completely disagree* |
| … Akinesia/rigor/joint constraints | ○ | ○ | ○ | ○ |
| … Cataract/physical impairments in the context of medication intake | ○ | ○ | ○ | ○ |
| … Hearing impairment/hearing loss | ○ | ○ | ○ | ○ |
| … Tremor | ○ | ○ | ○ | ○ |
| **D – Understanding for older patients** | | | | |
| Working on the following topics **enhanced my understanding for older patients**: | *I completely agree* | *I rather agree* | *I rather disagree* | *I completely disagree* |
| … Akinesia/rigor/joint constraints | ○ | ○ | ○ | ○ |
| … Cataract/physical impairments in the context of medication intake | ○ | ○ | ○ | ○ |
| … Hearing impairment/hearing loss | ○ | ○ | ○ | ○ |
| … Tremor | ○ | ○ | ○ | ○ |
| **E – Interest in working with older patients** | | | | |
| Working on the following topics increased **my interest in working with older patients:** | *I completely agree* | *I rather agree* | *I rather disagree* | *I completely disagree* |
| … Akinesia/rigor/joint constraints | ○ | ○ | ○ | ○ |
| … Cataract/physical impairments in the context of medication intake | ○ | ○ | ○ | ○ |
| … Hearing impairment/hearing loss | ○ | ○ | ○ | ○ |
| … Tremor | ○ | ○ | ○ | ○ |
| **Open questions** | | | | |
| **Please briefly specify your main insights from the online aging simulation course:** | | | | |

**Thank you for your support!**
